# Why axis inversion? Optimizing interactions between users, interfaces, and visual displays in 3D environments

**DOI:** 10.1186/s41235-025-00626-5

**Published:** 2025-06-23

**Authors:** Jennifer E. Corbett, Jaap Munneke

**Affiliations:** 1 Computer Science and Artificial Intelligence Lab, Massachusetts Institute of Technology, Room 32-D426, 32 Vassar St, Cambridge, MA 02139 USA; 2 Center for Cognitive and Brain Health, Northeastern University, 805 Columbus Ave, Boston, MA 02120 USA

**Keywords:** Y-axis inversion, 3D gaming, Stimulus–response-effect compatibility, Human computer interaction, Spatial orientation, Virtual environments, Control mapping

## Abstract

From video games to laparoscopic surgeries, differences in users’ abilities to adapt to new control schemes can have significant, even deadly impacts on performance. Starting with the question of why some video game players invert the y-axis on their console controllers, this work aims to provide a foundation for future investigations of how control schemes can significantly impact performance. We argue that fragmented research across disciplines hinders a unified understanding of how the spatial relationships between users, interfaces, and visual displays affect performance. Therefore, we begin with a multidisciplinary literature synthesis, clarifying existing findings, and identifying methodological inconsistencies that contribute to conflicting results. We then explore the relationship between key behavioral and cognitive factors and y-axis inversion preference in a group of experienced 3rd person gamers. Based on these preliminary results, we propose a “general purpose” framework to systematically investigate how control inversion and visual input influence perception and performance across various movement goals. We demonstrate how this framework can be used to evaluate performance in the context of a common and challenging laparoscopic procedure, and how it can be generalized to assess and predict sensorimotor compatibility effects across a wide variety of real-world situations.

**Supplementary Information:**

The online version contains supplementary material available at 10.1186/s41235-025-00626-5.

## Significance statement

This project exemplifies use-inspired basic research by addressing a real-world problem: the impact of inconsistent control schemes on user performance. These inconsistencies can lead to errors with potentially serious consequences. Despite its multidisciplinary relevance, research on this topic is fragmented. The lack of a unified approach hinders our understanding of how users interact with 2D interfaces representing 3D environments. Our work aims to bridge this gap. We begin by synthesizing research across relevant fields, clarifying existing knowledge, explicating methodologies, and identifying inconsistencies. Next, we explore the link between y-axis inversion preference and user traits in gamers. Finally, based on these findings, we propose a generalizable framework for investigating how control schemes and visual input impact performance in various real-world scenarios. Being able to learn and predict how a person will interact within a given environment can bring about monumental advancements to improve user experience and increase safety and efficiency. We hope that this work serves as a foundation for many future investigations of user-inspired, real-world problems and helps to bridge the gap between academic and applied knowledge

## Introduction

**Fig. 1:**
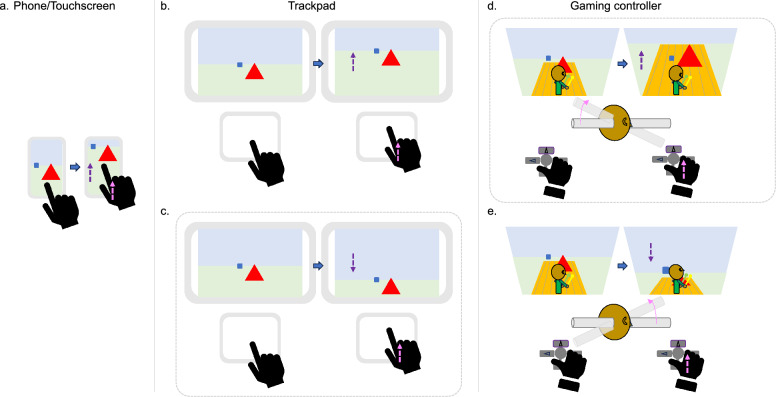
Different interaction styles with touch screens, trackpads, and gaming controllers. For each scenario, the left image shows the initial status, and the right image after the blue arrow shows the updated status after the user’s action. The magenta arrows indicate the direction of the user’s movement and the purple arrows indicate the corresponding direction information moves in the display. The dashed gray outline rectangles denote “inverted” control schemes. Whereas touch screens exhibit a natural swiping motion of information in the same direction as the user’s limb **a**, users’ preferences vary for scrolling on trackpads. Some opt for "natural scrolling" **b** of content in the same direction as the limb and others choose an inverted mode **c** where content moves opposite the limb. This inversion is not analogous to control configurations in 3rd person games, where "inverted" **d** involves the screen moving with the user’s limb as if moving the camera view or the avatar’s head from the back, and "non-inverted" **e** means the image moves opposite the user’s limb as if moving the avatar’s gaze with limb direction or the avatar’s head from the front

The world of gaming has witnessed significant advancements in the past 30 years. Game studios have built intricate 3D environments that are expected to become even more sophisticated as technology evolves. Successful interactions within such environments require cognitive and behavioral abilities that can differ greatly between individuals. In the gaming world, a hot button topic is whether players invert the y-axis when controlling the view in 3rd person games, where camera and avatar movements are partially separated (Stuart, 2020a; b; c). In most 3rd person games, pushing the game controller’s right joystick^1^ up makes the avatar look upward (Fig. 1e). This default scheme acts as if the user is moving an imaginary rod from the front of the avatar’s head, as a biologist moves slides beneath a microscope. Y-axis inversion is a user-customizable setting that reverses this control scheme. Pushing the joystick up makes the camera move upward (Fig. 1d), as if the user was moving an imaginary rod upward from the back of the head, like an astronomer moving a telescope over the stationary starry sky.

The debate over y-axis inversion has persisted, likely due to individual differences and varying gaming experiences. Importantly, multiple literatures in human factors, ergonomics, cognitive science, industry, and medicine all touch on issues relevant to y-axis inversion preferences. However, discrepant or even contradictory terminologies and discordant dissemination methods impede a unified view of the overarching problem space. A clearer cross-disciplinary understanding of the factors underlying inversion preference can serve as a starting point for future empirical investigations in a variety of situations. For example, although the option to invert controller axes mappings has become standard in most modern-day video games, real-world control mappings in safety critical situations like laparoscopic surgery may not allow for user customization. Toward a generalized understanding of why inversion preference varies so strongly between individuals, we first contextualized the problem over several existing literatures. We give special attention to methodological details for the sake of clarifying discrepant results and facilitating future work. This interdisciplinary review then serves as a foundation for our subsequent exploratory study of the relationship between y-axis inversion preference and key behavioral, cognitive, and user experience factors. Based on present and previous results, we conclude with a proposed method for systematically investigating how inverting controls and visual input influence perception and performance depending on movement goals. We then demonstrate how our framework can be translated to numerous real-world situations with individual and multiple users.

### What is Y-axis inversion?

Figure 1 compares the ways users typically interact with touch screens (e.g., smartphones), trackpads (e.g., laptops), and gaming controllers. Unlike touch screens where it is natural to swipe your finger in the direction you want the information on which you are acting to move (Fig. 1a), the preferred scrolling direction on a computer keyboard, mouse, or trackpad is not as straightforward. Some users maintain “natural scrolling” for moving the image on the screen in the same direction as the user’s controlling limb (e.g., finger) moves on the trackpad (Fig. 1b). Yet, others prefer to move the “viewing window” over the image, moving the image on the screen in the opposite direction of the user’s limb (Fig. 1c). The latter mode could be considered inverting both x- and y-axes. As illustrated in Fig. 1d&e, this is not directly analogous to control configurations in 3rd person games. Here, “inverted” (Fig. 1d) instead refers to a mapping where information on the screen moves with the user’s limb and the viewing window moves opposite (like the non-inverted trackpad setting; Fig. 1b), versus “non-inverted” (Fig. 1e) where the image moves opposite the user’s controlling limb (like the inverted trackpad setting; Fig. 1c). Importantly, users are typically meant to interact two-dimensionally with phone and laptop screens, whereas console games are usually intended to be perceived as 3D interactive environments. In console games, the illusion that the avatar is moving through the 3D environment is created in part by moving information on the screen around a stationary avatar (and user).^2^

**Fig. 2:**
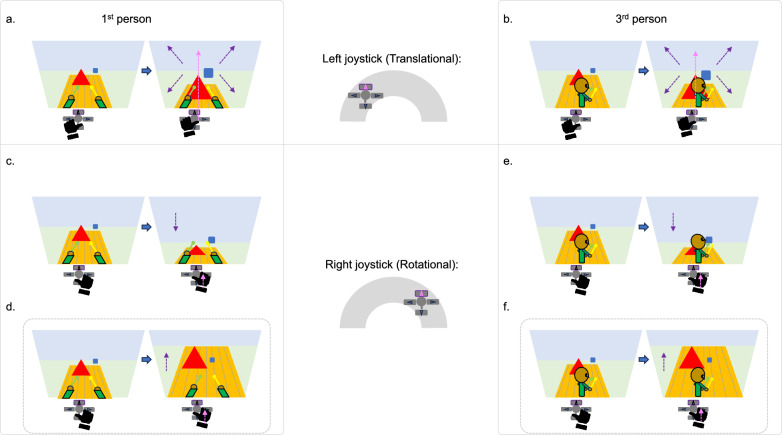
1st person (left) and 3rd person (right) scenarios for left joystick translational (top) and right joystick rotational (bottom) movements in console games. For each scenario, the left image shows the initial status, and the right image after the blue arrow shows the updated status after the user’s action. The magenta arrows indicate the direction of the user’s movement and the purple arrows indicate the corresponding direction in which information moves on the visual display. The dashed gray outline rectangles denote “inverted” control schemes. For translational movements in 1st person **a** and 3rd person **b** perspectives, moving the left joystick up moves the avatar forward and information against the user’s limb. For rotational movements, users tend to retain the default scheme **c** and rarely invert the y-axis **d** on the right joystick in 1st person games where the user views only the environment. In 3rd person games where the user typically views both the avatar and the environment, users more often switch from the default scheme **e** and invert the y-axis **f** on the right joystick

**Fig. 3:**
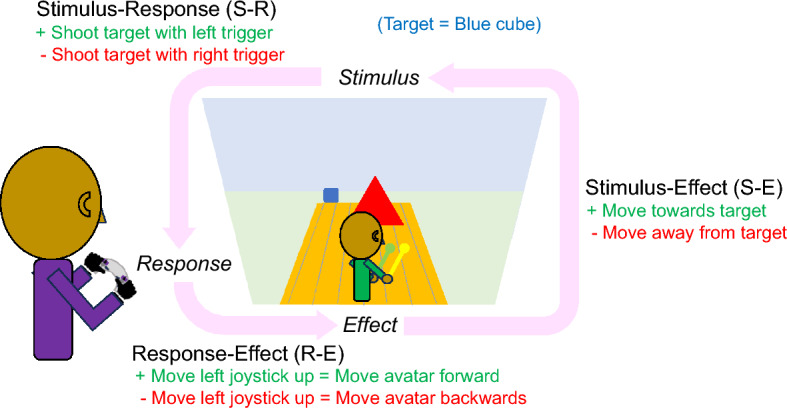
Possible relationships between the visible stimulus, the response action, and the effect this action has on the environmental stimulus. Stimulus–response (S-R) compatibility: If the blue (target) cube appears on the left side of the screen and the user is required to respond by pressing either the left or right trigger on the controller, there is S-R compatibility when the same side left vs opposite side right trigger is used for the response. Response-effect (R-E) compatibility: If the user needs to “go forward” using the left joystick, there is R-E compatibility when a “push forward” (up) motion of the joystick corresponds to the perception of forward movement. Stimulus-effect (S-E) compatibility: There is S-E compatibility if the avatar moves toward vs away from the blue cube

In addition to the extra dimension of depth, 3D console games can also differ in terms of the user’s 1st or 3rd person perspective (Fig. 2). A typical gaming controller has two joysticks: a left joystick for translational movement of the avatar’s body through the virtual environment (Fig. 2, top), and a right joystick for rotational movement of the avatar’s view and/or camera, largely independent of translational movement (Fig. 2 bottom). Translational forward, and sideways “strafing,” or turning movements are relatively straightforward. Regardless of 1st (Fig. 2a) or 3rd person (Fig. 2b) perspective, moving the left joystick “up” generally means “go forward,” such that the image on the screen moves against the user’s controlling limb. This aligns with the optic flow of information experienced when moving forward in the “real” environment, creating the perception that the avatar moves forward in the same direction as the user’s limb. Y-axis inversion mainly concerns how users employ the right joystick for rotational movements like surveying the environment and focusing on target locations. Although some also invert the y-axis on the right joystick in 1st person games where the user views only the environment (as the avatar; Fig. 2d), users typically retain the default, non-inverted configuration (Fig. 2c). The present discussion of y-axis inversion is focused on 3rd person games, where the user typically views both the avatar and the environment from an elevated perspective slightly behind, and off to the side of the avatar’s shoulder (Figs. 2e-f).

Although our investigation of y-axis inversion concentrates on 3rd person games, the option to invert originated in 1st person (shooter) games. To our knowledge, Quake (id Software, 1996) was the first widely played game to offer players the option of changing the view in multiple directions, using the mouse as if to move the avatar’s head (referred to as “mouselook”). Before this, the only commercially popular platform that employed an inverted y-axis scheme (by default) was Microsoft’s Flight Simulator series (Microsoft, 1982). Halo: Combat Evolved (Bungie, 2001) was the first major game to tackle the challenge of determining players’ y-axis mapping preferences by integrating an in-game “optical diagnostic station,” where players used the controller to “look” at different points on the screen. Iterations of these diagnostics are still used to aid players in customizing their controllers. However, there is currently no publicly available, empirically validated method for determining controller mapping preferences, nor has there been any systematic investigation of the cognitive and behavioral factors underlying inversion preferences.

### User experience

Despite the long-standing debate within the gaming community, there has only been one study of psychological aspects underlying y-axis inversion preferences. Frischmann et al. (2015) surveyed a group of 150 college students about their gaming habits, demographics, Part V of the Guilford-Zimmerman Aptitude Survey measuring spatial orientation abilities (Guilford & Zimmerman, 1948), and the Presence Questionnaire (PQ) and Immersive Tendencies Questionnaire (ITQ; Witmer & Singer, 1998). Presence refers to the subjective experience of physically being in an environment. Immersion is the more general sense of being engrossed or absorbed in interactive experiences. After completing the surveys, participants played the avatar response mapping portion of the tutorial for Sony PlayStation’s ‘Tom Clancy’s Ghost Recon 2’ (Red Storm Entertainment, 2004). After the tutorial, participants completed the NASA Task Load Index (NASA-TLX) (Hart & Staveland, 1988), a subjective workload measurement. Participants whose y-axis inversion preferences were mismatched with their randomly assigned game-play condition scored significantly higher on the perceived performance workload measure. Examining scores as a function of inversion preferences resulted in significant differences on the PQ and ITQ, as well as the Focus and Games ITQ sub-scales (but no significant differences on the ITQ Involvement sub-scale). Non-inverters scored significantly lower than always-inverters on the PQ and the ITQ Focus sub-scale, whereas non-inverters scored significantly lower than sometimes-inverters on the total ITQ score, as well as the ITQ Games sub-scale. Clearly, using non-preferred mappings affected how participants perceived the gaming experience. However, it is unclear why non-inverters would be less immersed. Non-inversion corresponds more to viewing the virtual environment *as* the avatar, whereas inversion is more analogous to controlling the avatar within the virtual environment. In any case, Frischmann et al. (2015) results point to presence and immersion as potential predictors of users’ y-axis inversion preferences.

### Which way is up?

Beyond user experience factors like immersion and presence, inversion preferences may also be linked to differences in how individuals rely on visual information to perceive “upright,” or the direction of the pull of gravity. We base our perceptions of upright on cardinal lines inherent in the visual environment to the extent that we treat even a single horizontal line as the “horizon,” and evaluate the orientation of surrounding objects with respect to this simple reference. When the visual environment is tilted, we misperceive upright in the direction indicated by visual references even when our bodies are aligned with the pull of gravity. This was first noted by Asch and Witkin (1948) who had observers adjust a rod inside a tilted room to vertical. Their judgments of vertical consistently erred in the opposite direction of the tilted room (the “Rod-and-Frame Illusion”). With prolonged exposure, observers eventually perceived the tilted room as upright and felt as if they were tilted (e.g., Passey, 1950).

Witkin et al. (1977) outlined two classes of observers with respect to how they use visual and bodily cues to determine upright. Even though all observers misperceived upright when viewing a tilted frame, some participants consistently demonstrated less bias. These “field independent” observers tended to use internal cues or body referents, whereas “field dependent” observers tended to use external “field” references for upright. Hecht and Reiner (2007) later found that field dependence negatively correlated with participants’ scores on a presence questionnaire (adapted from Witmer & Singer, 1998). Importantly, a controller’s y-axis is oriented relative to the upright visual environment and the player choses whether to invert the joystick to interact with this information. Consequently, the extent to which gamers depend on the orientation of the visible environment may be an important predictor of inversion preference, and this field dependence may also covary with the experienced sense of presence.

### Cognitive spatial abilities

Findings discussed so far suggest that inversion preferences could stem from psychological and perceptual factors, or could be innate due to environmental or evolutionary factors. Individual preferences might also be more broadly linked to cognitive spatial abilities like mental rotation and perspective taking. Mental rotation requires the observer to imagine spatially transforming an object. Perspective taking requires mentally changing one’s own position within an external frame of reference. In the context of y-axis inversion, mental rotation maps on to the ability to make sense of the movements of the controller when navigating through the virtual environment. Perspective taking is directly related to the interaction between different avatar and camera perspectives when selecting an appropriate view for the task at hand.

Findings from behavioral studies point to distinct abilities to make transformations of objects and transformations of an observer’s position within an environment. Shepard and Metzler (1971) first reported that the time it takes people to determine whether two drawings represent the same 3D shape has a linear relationship with the angle of physical rotation. This suggests observers are mentally rotating the mental image analogous to physically operating on the corresponding real-world object. Kozhevnikov and Hegarty (2001) separately assessed observers’ perspective taking abilities by presenting them with arrays of objects and measuring their angular errors when taking on a given perspective and indicating the direction of a target from this perspective. Kozhevnikov et al. (2006) demonstrated a unique predictive value for perspective taking ability in anticipating performance in real-world navigation tasks. After completing a perspective taking task (Kozhevnikov & Hegarty, 2001) and a mental rotation task (Shepard & Metzler, 1971), participants followed the experimenter on a route over two floors of an unfamiliar building and completed subsequent navigational tasks. Perspective taking and mental rotation accuracy both correlated with their abilities retrace the route on floorplans. However, only perspective taking accuracy correlated with their abilities to indicate the direction to two landmarks along the route and to find a shortcut from the end to the start of the route. Collectively, these results suggest that gamers’ performance in perspective taking tasks may be more predictive of their inversion preferences than performance in mental rotation tasks.

### Sensorimotor compatibilities

Dissociable abilities in mentally transforming an object’s position versus one’s own position point to differences in how users manipulate information within displays compared to how they interact within the visible environment. In addition to these cognitive abilities, there are three main types of spatial sensorimotor compatibilities that generally govern how users interface with displays and controllers. Stimulus–response (S-R) compatibility, response-effect (R-E) compatibility, and stimulus-effect (S-E) compatibility are concepts used in human–computer interaction and psychology, particularly in the context of designing interfaces. As illustrated in Fig. 3, if a stimulus (e.g., blue cube) appears on the left side of the screen and the user is required to respond by pressing either the left or right trigger on the controller, there is S-R compatibility (+ S-R) when the same side left trigger is used for the response. A response using the opposite side right trigger would be incompatible (-S-R). If the user needs to “go forward” using the left joystick, there is R-E compatibility (+ R-E) when a “push forward” (up) motion corresponds to the perception of forward movement. When an upward motion of the joystick corresponds to the perception of backward avatar movement, the controller is spatially incongruent with the actions of the avatar (-R-E). Finally, there is S-E compatibility if the avatar moves toward the blue cube stimulus (+ S-E) as a result, and S-E incompatibility (-S-E) if the user moves away from the blue cube.

*S-R Compatibility:* Stimulus–response (S-R) compatibility has been studied extensively. In one of the first investigations, Fitts and Seeger (1953) tested how the overlap between the spatial layout of a stimulus and response affected Air Force pilots’ performance. They observed a strong interaction between the layouts of stimulus light panels and response panels of pathways that pilots traced with a stylus. Performance was best when each stimulus panel (e.g., an array of two horizontal lights on the left and two vertical lights on the right; **— |**) was paired with the most spatially corresponding response panel (e.g., separate pathways for left and right movement by the left hand and up and down movements by the right hand; **— |** versus four overlapping pathways; ${ \boxplus }$). In a subsequent study, Fitts and Deininger (1954) found that congruent S-R pairings resulted in the best performance compared to mirror image pairings or random pairings. S-R compatibility dominates responses even when the stimulus location is task irrelevant. Simon and Wolf (1963) demonstrated that when the color of a right light was associated with a right button press (+ S-R), participants made faster correct responses than when the correct response was linked to a left button press (-S-R; i.e., “The Simon Effect”). Together, results suggest performance is superior when there is direct transfer between stimulus and response to maximize congruency and minimize the number of “recoding” steps.

Second-order S-R compatibility effects can also be implicitly related to perspective taking. The magnitude of the Simon Effect can index the extent to which users are able to take on an avatar’s perspective (i.e., overcome first-order -S-R effects of an inverted visual display). For example, Müsseler et al. (2019) presented participants with a virtual avatar, either aligned with their point of view or rotated 180° so the avatar faced the participant. When taking on the avatar’s perspective and responding to colored disks with a left or right side button, participants displayed “Simon Effect” patterns of performance relative to the avatar’s versus their own perspective. Importantly, responses were generally faster and more accurate when the participant’s perspective was the same as the avatar’s, indicating some extra cognitive processing associated with first-order -S-R effects when taking the avatar’s opposite perspective. Given that S-R compatibility affects performance even when spatial location is task irrelevant, these findings further suggest S-R compatibility and perspective taking may be important and covariate predictors of y-axis inversion preference.

Importantly, controls are generally designed with specific stimuli in mind. Gaming controllers are designed to map onto the content displayed on screen moreso than screen content is designed to map onto controllers, especially as there is relatively little control over the layout of the natural environment video games often aim to simulate. Whereas S-R compatibility is largely dictated by the stimulus, the relationship between how the controlling mechanism moves and the effect this has on the movement of the stimulus (R-E compatibility) is not as clear-cut.

*R-E Compatibility:* The mapping between the direction the controller moves and the direction this moves the stimulus of interest, or response-effect (R-E) compatibility is directly related to gamers’ preferences for y-axis inversion. As mentioned, R-E compatibility in gaming is more ambiguous for rotational right joystick movements versus translational left joystick movements. For translational movements, R-E compatibility refers to the motion of the joystick (e.g., “forward” or “up”) resulting in a corresponding directional perception of the avatar moving (e.g., forward) through the virtual environment. On the one hand for rotational movements, the command “look up” could be mapped as an upward movement of the right joystick that corresponds to the avatar’s gaze moving up. In this case, the controller is spatially congruent with the actions of the avatar (+ R-E). On the other hand, the command “look up” could be mapped to the controller as a downward movement of the right joystick corresponding to “move avatar’s head backwards,” or “move the camera down.” Now, movement of the controller is spatially congruent with the direction the virtual environment is moved on the screen but incongruent with avatar’s actions (-R-E).

To date, there have been no studies of R-E compatibility for rotational movements on gaming controllers. There are some related findings from ergonomic and human factors experiments. For example, Worringham and Beringer (1989) asked people to move a cursor to a target using a joystick and varied the position of participants’ bodies and/or the joystick relative to the display to independently manipulate compatibility between 1) the visible direction the participant’s hand moved and the direction the cursor moved (visual field compatibility), 2) the direction the joystick moved and the cursor’s movement (control display compatibility), and 3) the movement of the participant’s wrist (flexion or extension) and the cursor’s movement (muscle synergy compatibility). Participants made overall faster and more accurate movements when visual field compatibility was achieved regardless of control display or muscle synergy compatibilities.

Findings by Kunde (2001) suggest that anticipation is another key factor underlying R-E compatibility effects. Participants pressed one of four keys in a row, assigned to the left and right index and middle fingers to discriminate the color of a centrally presented dot. After the participant responded, one of four squares above the four response keys turned white. In the corresponding condition (+ R-E), the square above the correct response key turned white immediately after the key was pressed. In the non-corresponding condition (-R-E), response mappings were flipped to the same alignment as physically crossing the hands. Participants responded faster in the corresponding condition even though the square did not turn white until after they responded. These results suggest that R-E compatibility is largely a product of the agreement between the visible body part making the response and the expected effect on the visible stimulus.

Results discussed so far suggest that moving the stimulus in the same direction as the body maximizes performance along the horizontal, left–right x-axis pointing to x-axis inversion preferences as a potential predictor of gamers’ y-axis preferences. However, there is a mixed bag of results from studies of scrolling behavior and y-axis R-E compatibility. In an early study, Bury and colleagues (1982) presented participants with a centrally aligned cross stimulus consisting of sequential letters along the vertical meridian and sequential numbers along the horizontal meridian. Participants used the arrow keys to navigate to a letter and/or number target that was initially out of view. Participants in “window” (-R-E; inverted y-axis) groups used key mappings corresponding to moving a window over the data where pressing “up” displayed data toward the top of the stimulus (start of the alphabet). Participants in “scroll” (+ R-E) groups used key mappings corresponding to moving the data so that pressing “up” displayed data toward the bottom (end of the alphabet). Inverted (-R-E) conditions led to better performance with faster response times and fewer moves in the ‘window’ groups, and significantly more participants self-selecting the “window” mapping.

Importantly, Bury et al.'s (1982) study was conducted in the early 1980s, when digital displays were not as widely used as they are today and smartphones did not exist. Scrolling direction became a hot topic in 2011 when Apple introduced Mac OS X Mountain Lion (OS X 10.7), due to the novel default setting of “natural scrolling,” where content swipes with finger movement (Fig. 1b), like modern touchscreens (Fig. 1a). Chen and Proctor (2013) tested how manipulating the response mapping between the arrow keys and the movement of stimuli on the screen affected participants’ performance. As in Bury et al.'s (1982) study, participants completed two response mapping conditions using the arrow keys to move a circle or number target into view. In the + R-E condition, the screen moved in the direction of the arrow, such that pressing “up” showed the stimulus on the bottom of the screen. In the -R-E condition, screen content moved in the opposite direction, such that pressing “up” uncovered the stimulus on the top of the screen. Contrary to Bury and colleagues’ (1982) findings, participants were significantly faster in + R-E condition where the screen content moved with arrow direction.

These opposite patterns of results are likely due to monumental technological advancements during the 31-year period between studies. Participants in the later study had considerable everyday experience using desktop computers, laptops, smartphones, trackpads, and tablets, whereas participants in the former study probably had minimal experience with desktop computers. Along these lines, gamers often cite experience with early gamified flight simulators and their familiarity with inverting since they began gaming (e.g., whether they played their first game and/or all-time favorite game with an inverted configuration, whether they used a specific gaming system) as reasons for their y-axis inversion preferences. Therefore, in addition to basic demographic factors (age, handedness, and gender), how long gamers have been playing 3rd person games, the first, most recent, and favorite games they have played, the type of console(s) they use, and even their preferences for trackpad scrolling direction may be important predictors.

*S-E Compatibility:* Stimulus-effect (S-E) compatibility has been studied the least, likely because most investigations are concerned with directing actions to (+ S-E) versus avoiding (-S-E) stimuli. Furthermore, S-E compatibility is often confounded with S-R and/or R-E compatibility. In Kunde et al. (2007), participants moved a lever controlling a tool that either moved with (+ R-E) or against (-R-E) their hand, toward (+ S-E) or away from (-S-E) a target. There was a main effect of R-E compatibility with faster responses when the lever moved with versus opposite the hand, a main effect of S-E compatibility when the lever moved toward versus away from the stimulus, but no interaction suggesting independent effects of R-E and S-E compatibility. However, S-R compatibility was confounded, such that R-E and S-E compatibilities were always matched in + S-R conditions where the hand and stimulus were on the same side.

Müsseler and colleagues (2008) decoupled S-R and S-E compatibilities using a T-shaped lever moved around a central pivot point. Performance differenced as a function of whether participants used a grip at one end to move the tip toward versus away from a target (S-E compatibility), and whether that target was on the same side as the grip (S-R compatibility). Faster correct responses were made when S-R and S-E matched (+ S-R + S-E and -S-R -S-E) versus mismatched. However, S-R and R-E were now confounded, as the hand was always on the same side as the target (+ S-R) when the tool tip moved in the same direction as the hand (+ R-E).

Müsseler and Skottke (2011) used a U-shaped lever and an inverted-U lever to independently manipulate S-R, R-E, and S-E compatibilities. Participants moved the lever to either contact (+ S-E) or avoid (-S-E) a target, and the target could be on the same (+ S-R) or the opposite side (-S-R) of the screen as the participant’s controlling hand. The inverted-U lever was used in conditions with + R-E compatibility, such that the tip of the tool moved in the same direction as the hand movement. The U lever was used in -R-E conditions where the tip of the tool moved in the opposite direction as the hand. Overall, main effects of R-E and S-E revealed that performance was better when the tool and hand moved in the same (+ R-E) versus opposite (-R-E) direction, and when the tool was moved toward (+ S-E) versus away from (-S-E) the stimulus. However, there was only a benefit of having the hand and the stimulus on the same side (+ S-R) when S-E and R-E were also compatible.

Taken together, results suggest an interactive relationship between S-R-E compatibilities. Given the variation in stimuli and the extent to which they translate to a given real-world task, it is difficult to draw more precise conclusions about the relative contributions of each type of compatibility. Although results collectively point to benefits of compatibility over incompatibility, studies conducted decades apart suggest preferences may not be innate. Users may quickly adapt to incompatible or novel control schemes. As next discussed, inversion preferences may be domain specific and even reverse with expertise.

### Gaming as training

Although inversion and spatial compatibility effects are not apparent safety threats to gamers, video games are increasingly being used for simulated training in safety–critical real-world situations where control customization is not always possible. For example, “Nintendo surgery” is a widely used method for training or “warming up” surgeons for endoscopic procedures that are more cognitively demanding (e.g., Satava, 1992). Laparoscopic surgery is inherently faced with the issue of inverting the x- and y-axis. By default, there is -R-E incompatibility (widely known as “the fulcrum effect”). Because the instruments pivot around the port of entry, the direction a surgeon moves an instrument outside the patient’s body is opposite the direction the tip of the instrument moves inside the body. To make matters even more complicated, laparoscopic surgeries that involve two surgeons on the opposite sides of a patient’s body restrict the perspective of the internal camera to only one of the surgeons. Unless the display can be corrected for the other surgeon, they are challenged with a -S-R “paradoxical"view^3^” of a left–right, up-down reversed image.

The existing literature largely addresses these issues independently, with mixed results for each. Some findings point to benefits of + R-E in “correcting” the -R-E fulcrum effect. Others suggest there is no benefit or even detrimental effects of + R-E from inverting the image to align with the direction the surgeon moves the instrument. Gallagher et al. (1998) evaluated the abilities of naïve participants to perform a paper cutting task designed to mimic laparoscopic surgical conditions with normal (-R-E) or inverted (+ R-E) viewing conditions. Participants moved laparoscopic forceps and scissors left and right to make incisions on a sheet of paper presented within a laparoscopic training unit. One group viewed the image normally with the fulcrum effect (-R-E). The other group viewed the image inverted around the y-axis (essentially inverting the x-axis^4^), such that the working end of the instrument moved in the same direction as the external end (+ R-E). Although both groups improved over time, participants in the inverted (+ R-E) condition had an initial advantage and learned the task faster. These findings align with results from Kunde et al. (2007) to suggest that realigning the direction in which the tool, hand, and controller move to “correct” the fulcrum effect may also be beneficial in the context of laparoscopic procedures.

It is important to note that both studies tested participants with no previous experience operating laparoscopic equipment. When experienced surgeons performed the same paper cutting task used in Gallagher et al., (1998), they demonstrated superior performance under laparoscopic (-R-E) viewing conditions (Crothers, et al., 1999). Indeed, results from gaming simulations suggest that novice surgeons can be trained to overcome performance challenges related to the fulcrum effect (e.g. Bokhari et al., 2010). Taken together, these results suggest that -R-E fulcrum effects are easily overcome in laparoscopic surgery. However, results cannot be generalized across domains and tasks, as directly apparent when changing scrolling direction on your current trackpad settings or selecting the opposite of your usual preference when setting up the controller in a 3rd person game.

In addition to the fulcrum effect, laparoscopic surgeries sometimes involve inserting the camera on the opposite side of one of the surgeons, resulting in a left–right up-down reversed camera image (paradoxical view, -S-R), as if looking from the camera operator’s perspective while physically positioned facing the operator. Johnston et al. (2003) first reported that both experienced and novice surgeons completed cutting and transferring tasks in a laparoscopic simulator faster with the camera image inverted about the x- and y-axes (+ S-R) versus under paradoxical viewing conditions (-S-R). Whereas novices can rapidly overcome the -R-E compatibility of the fulcrum effect (a mapping preferred by experienced surgeons), substantially longer training is needed to reach surgical competence with -S-R paradoxical viewing. Hwang et al. (2010) followed three fellows at the start of their laparoscopic training in assisting colorectal surgeries where they were positioned with paradoxical (-S-R) camera view. Learning curves for grasping tissue and the number of grasp attempts over a procedure gradually decreased with surgical experiences, asymptoting between 30 and 40 procedures before competence was reached (See also, Gould & Frydman, 2007). Minor improvements in performance have been reported from placing the monitor at different positions relative to the viewer (e.g., Haveran, et al., 2007), or partially rotating versus inverting the image (e.g., Inagaki, et al., 2021). However, there are no straightforward links to gamification training helping novice surgeons overcome the -R-E fulcrum effect. Taken together, historical influences on gamers’ y-axis inversion preferences and domain-specific, experience-dependent effects of R-E compatibility pose significant challenges for a clear understanding of how it relates to gamers’ self-reported inversion preferences.

### Present study

Based on this integrated review, we next conducted an exploratory investigation of the relationship between 28 key user experience, behavioral, and cognitive factors and experienced 3D console gamers’ inversion preferences. A questionnaire was used to examine user and gaming factors. We adopted the Presence Questionnaire (PQ) and Immersive Tendencies Questionnaire (ITQ; Witmer & Singer, 1998) as standard measures of user experience. In addition to typical demographic information such as age, gender, and handedness, we developed a custom survey of other user factors highlighted in the literature synthesis (see Supplementary Materials). Along with questions regarding y-axis inversion preferences in 3rd person games, we also inquired about x-axis inversion preferences, and trackpad scrolling preferences. Given how historical factors may influence control mapping preferences, we also asked participants about what games and gaming systems they used and preferred over time.

To examine how some of the main cognitive factors reviewed might predict y-axis inversion preferences, we included four computerized tasks; a field dependence (FD) task to assess the extent to which an individual perceives “up” as a function of the immediate visual context, a mental rotation (MR) task to measure the efficiency (speed and accuracy) with which participants could mentally manipulate objects, a Perspective Taking (PT) task to assess how quickly and accurately individuals were able to take on discrepant perspectives, and a response mapping (RM) Simon task as an initial assessment of how S-R compatibility affects individuals’ performance.

By quantifying the key factors highlighted in the literature, we can then examine the relative importance of each in predicting an individual’s y-axis inversion preference. Results can inform our understanding of why gamers might invert, as well as provide an overview of potentially relevant factors for more general investigations of inversion preferences across multiple domains.

## Methods

*Participants:* Participants were recruited via a news article in *The Guardian* (Stuart, 2020b) concerning the topic of y-axis inversion. All participants (*N* = 192) were invited to fill in an online survey and subsequently partake in a series of online experiments. Attrition rate was approximately 11.5%, as 22 participants were replaced or dropped from the dataset for varying reasons, ranging from filling in the survey but not completing the full set of experiments to experiencing technical problems with the online experiments. Only the remaining 170 complete datasets were used for further analysis. All participants self-reported normal or corrected-to-normal vision and were between the ages of 18 (age of consent) and 35 (approximately the age when visual acuity tends to initially decline due to the onset of presbyopia). All participants provided online informed consent in accordance with the Declaration of Helsinki, and all aspects of the study were approved by Brunel University London’s Institutional Review Board.

*Procedure & Materials:* Participants first completed a single questionnaire including versions of the presence questionnaire (PQ) and the immersive tendencies questionnaire (ITQ; Witmer & Singer, 1998) as well as questions related to their demographics and gaming habits (see Supplementary Materials), followed by four behavioral experiments (the order of which was counterbalanced over participants). The study was conducted in a hybrid manner with participants completing the questionnaire and experiments online on their laptop or desktop computer (no tablets or phones were permitted). The experimenter was present via video-conferencing software (Zoom) on the participant’s phone or tablet so they could instruct and observe the participant’s performance and behavior, recreating a minimal but adequate level of lab-based control. At the beginning of the session, the experimenter emailed the participant a link to the questionnaire created with Google Forms and instructions to follow the link and complete all questions in the questionnaire. After the participant had completed the questionnaire, the experimenter sent them a subsequent email (during the ongoing session) with the links to the four experiments in the specific counterbalanced order for the given participant.

All experiments were conducted online using a standard chromium-based browser through Pavlovia (pavlovia.org) implementing Python-based scripts converted to JavaScript, created in PsychoPy (Peirce et al., 2019). To ensure that stimulus displays were comparable on the various devices used by the large group of participants, we used PsychoPy’s “normalize” function to calculate size and position of the various stimuli, relative to the size of the participant’s screen. Using this function resulted in participants having relatively comparable displays, independent of screen size and resolution. Throughout this section, stimulus size and position will be discussed as relative size (*rs*; in percentages). Here, *rs* is expressed as relative to the width of a screen with a 16:10 aspect ratio.^5^ This method partially circumvents the lack of control over individual participants’ test setups. Combined with the within-subjects design of the experiments, this approach yielded a testing environment sufficient to produce reliable and comparable data.

Participants were instructed to sit at approximately arm’s length from their laptop or computer screen, and the experimenter ensured they remained in this position throughout the duration of each experiment. Each experiment began with on-screen instructions followed by a short practice block to better ensure participants understood the task*.* None of the practice data was used in any of the subsequent analyses. Participants took approximately 10 min to complete the questionnaire, and between 15 and 30 min to complete each of the four subsequent experiments, depending on the amount of practice required to completely understand and execute the task.

Importantly, the questionnaire provided an opportunity for participants to make open-ended statements about their gaming habits related to y-axis inversion. Many participants attributed their y-axis inversion/non-inversion preferences to playing Microsoft Flight Simulator (many non-participants also emailed the authors with this opinion). In addition, many participants anecdotally reported changing their inversion preferences over time. Given the importance of historical factors in the scrolling and laparoscopic literatures, we contacted participants once more via email to inquire whether they had ever regularly played Flight Simulator, and whether their y-axis inversion preferences had changed over time. In total, 143 out of the 170 participants responded to these two additional questions.

**Fig. 4:**
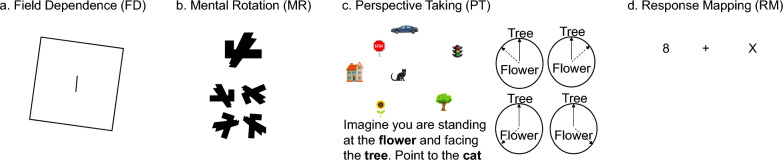
**a** Field Dependence (FD) task: On each trial participants saw line surrounded by a tilted square and their task was to indicate whether the line was tilted to the left or right. The correct answer in this example is “right.” **b** Mental Rotation (MR) task: On each trial participants saw a target shape and four possible choices. Their task was to select the item that was a rotation of the target shape. The correct answer in this example is the shape on the bottom right. **c** Perspective Taking (PT) task: On each trial participants were presented with instructions and four possible choices. Their task was to select the choice that corresponded to the perspective in the instructions. In this example, the correct choice is in the top left location. **d** Response Mapping (RM) task: On each trial participants saw a number on one side of the screen and an X on the opposite side of the screen and their task was to press the “E” key with the left index finger if the number was even or the “O” key with the right index finger if the number was odd. In this example, the correct answer is “E” for even

*Field Dependence:* The Field Dependence experiment (FD, Fig. 4a) was adopted from a previous similar RFI paradigm by Corbett et al. (2009). Each trial started with a white outline fixation circle (rs = 0.82%) centered on a gray (RGB 128, 128, 128) background for 500 ms. After fixation offset, a short black line (rs = 1.81%) centered within a black outline square (5 pt line widths, square side rs = 17.20%) was presented in the center of the screen for 100 ms. Using the method of constant stimuli, the square could be tilted ± 15° to the left or right, and the central line was tiled from −8° to + 8° in 2° steps from vertical, for a total of 18 possible combinations of square and line tilt. The square and line were immediately followed by a circular noise pattern mask (diameter: rs = 32.91%) for 100 ms to prevent confounding visual aftereffects. The screen then remained blank until participants responded. Participants’ task on each trial was to press the left arrow key if the line inside the square appeared tilted to the left and the right arrow key if the line appeared tilted to the right. They were instructed to respond as quickly and accurately as possible, but accuracy was stressed over response time. Each participant first completed 18 practice trials, during which the experimenter closely monitored their performance, gave feedback about the speed and accuracy of their responses, and clarified any of the participant’s questions. Participants then completed 180 experimental trails (10 trials of each of the 18 possible square/line tilt combinations in random order) with no feedback.

*Mental Rotation:* Each trial in the Mental Rotation task (MR, Fig. 4b) started with a black fixation cross (rs = 0.39%), presented on a gray (RGB: 128, 128, 128) background for 200 ms. Subsequently, a display containing one large abstract 2D shape presented on a white square (encompassing square side: rs = 19.74%) on the top of the screen, with four test shapes (each test shape presented on a white square with sides approximating a relative size of 13.16%) arranged in a 2 × 2 layout below this target shape. The participant’s task was to click on the test shape that corresponded to a rotation of the target shape, adapted from Ekstrom and colleagues’ (1976) Card Rotation Test. Rotation was defined as the one shape that could be accomplished by rotating the target shape in a 2D space, without having to “flip” the image over the horizontal or vertical axes. Participants were told that both speed and accuracy would be measured but to prioritize accuracy, and the target and test shapes remained on screen until the participant responded by selecting one of the four options. A short, 400 ms interval separated the trials.

A total of 25 unique target shapes were created, and four corresponding test shapes were created for each target shape. Correct choice test shapes were constructed by rotating the target shape either 45°, 135°, 225°, or 315° clockwise, determined randomly for each target shape. The remaining three test shapes for a given target shape were created by flipping the target shape about the vertical or horizontal axis and then applying one of the four possible rotations or no rotation, also randomly determined without replacement for each set of three incorrect choice test shapes. The same 25 sets of shapes were used for all participants, but the order of the sets over trials for each participant and the positions of the individual test shapes in each trial were fully randomized. Participants first completed five practice trials, during which they received feedback concerning the accuracy of their responses (for one second in the center of the screen after each practice trial). Following practice, participants completed 20 experimental trials with no feedback. Note that the 25 total shape sets were presented in random order without replacement over both practice and experimental blocks. A given shape set could be used only in practice for one participant and only in experimental trials for another participant, but the same set was never reused in any trial within a single participant.

*Perspective Taking:* The perspective taking (PT) task was adapted from the Object Perspective-Taking Test (Hegarty & Waller, 2004; Kozhevnikov & Hegarty, 2001). Following a brief fixation cross (rs = 0.39%; 200 ms) on a uniform gray background (RGB: 128, 128, 128), participants were presented with a set of seven colored objects on a white square background centered in the top half of the screen (Fig. 4c; rs = 27.96%); a car, a traffic light, a stop sign, a cat, a tree, a house, and a flower. They were also presented with written instructions to imagine themselves standing at one of the objects (e.g., the flower) facing another object (e.g., the tree), and to “point” at a third object (e.g., the cat). Participants used the mouse to click on one of four test circles arranged in a 2 × 2 layout below the display of objects to indicate the correct relationship (circle diameter: rs = 9.21%). For each of the four circle alternatives, the name of the object where the participant imagined standing was presented in the center. The name of the object they were to imagine facing was presented at the top of the circle with a continuous arrow drawn from the center to the top. A second dotted arrow was drawn from the center to a different location in each of the four test circle choices. Participants had to use the computer mouse to select the circle with the dotted arrow pointed toward the correct location where they were instructed to imagine pointing. The locations of the individual response options within the 2 × 2 layout were randomly determined on each trial. Options were presented simultaneously with the display of objects. The entire display remained on the screen until participants responded. Participants were instructed to respond as quickly and accurately as possible, with accuracy stressed over response time. They first began the task with two practice trials with feedback about accuracy (“Correct” or “Incorrect”) presented for 1 s after the offset of the display. Practice was followed by 10 trials with unique instructions, presented in random order. Each trial display was preceded by a small white fixation cross on an otherwise gray background. After participants responded on each trial, they were presented with a blank gray screen for 400 ms (ITI).

*Response Mapping:* For the Response Mapping (RM) task, we adapted a basic version of the Simon Task (e.g., Simon & Wolf, 1963). On each trial, participants were shown a display containing a fixation cross in the center of the screen (rs = 1.65%), flanked by a number (0–9), and the letter “X” on either side of the fixation (rs = 4.28% by 4.95%), at a distance of rs = 18.10% from the vertical edges of the screen (Fig. 4d). Participants were instructed to locate the number and respond to its parity (odd or even) as quickly and accurately as possible. All stimuli remained on the screen until a response had been made. Participants pressed the “E”-key with their left index finger when the number was even, and the “O”-key with their right index finger when it was odd. The combination of response mapping (left button, right button) and target location (left or right side of the screen) led to either spatially congruent or incongruent trials. On congruent trials (+ S-R), the stimulus appeared in a location that corresponded to the response side. On incongruent trials (-S-R), the stimulus appeared on the opposite side of the screen. Stimulus parity and location were counterbalanced, resulting in 50% congruent and 50% incongruent trials. The task consisted of four practice trials followed by 40 experimental trials. Feedback was provided during the practice phase (1 s), but not during the experimental trials. Each response was followed by a 200 ms inter-stimulus interval.

## Analysis & results

*Data Coding and Formatting:* Over the questionnaire and experiments, we measured 28 potential predictors of y-axis inversion identified in the literature synthesis. The entire set of formatted data used for our main factor-ranking analysis is available here: https://osf.io/3j6ds/.

From the demographics and gaming habits portion of the questionnaire, we categorized participants’ y-axis inversion preferences (InvertY) as Never = 1, Sometimes = 2, or Always = 3. We also categorized participants’ x-axis inversion preferences (InvertX) as Never = 1, Sometimes = 2, or Always = 3. Participants’ ages (Age) and the number of years they had been playing 3rd person console games (YearsPlaying) were input as continuous variables. Handedness (Handed) was categorized as Left = 0, Right = 1, Both/Ambidextrous = 2, Gender (Gender) was categorized as Female = 0, Male = 1, Nonbinary = 2, whether participants had ever switched their inversion preferences (Switch) and whether they had played flight simulator (Flight) were both categorized as No = 0, and Yes = 1. For the most recent 3rd person console game played (MostRecent3rd), first 3rd person console game played (FirstGame3rd), and favorite 3rd person game (Favorite3rd), we excluded any games that were not 3rd person (e.g., Halo), and standardized game titles (e.g., “TombRaider” was used regardless of any additional version information provided by the participant, such as “Tomb Raider 3”). We used the participant’s self-selected category for their favorite gaming genre (Genre). For each participant’s current console gaming system (Console), for each type of console the participant listed, we categorized their responses as No = 0, and Yes = 1 for each of four major console categories (Playstation, Nintendo, PC, Xbox). Given that most participants were currently using more than one type of system, we also coded a single 4-digit variable as the combination (Combo) of these systems, respectively (e.g., 0110 represents a participant with Nintendo and PC systems, but not Playstation or Xbox).

We also calculated five measures from participants’ Likert responses on the PQ (Presence) and ITQ (Immersion) sections of the questionnaire. First, we summed each participant’s responses over all 31 of the scale measures on the PQ (SumPresence; out of 217 possible total) and all 28 measures on the ITQ (SumImmersion; out of 196 total). We next calculated their ITQ sub-scores on the “Focus” (ITQFocus), “Games” (ITQGames), and “Involvement” (ITQInvol) sub-scales (out of 49, 14, and 49, respectively).

For the FD experiment, we first calculated the average proportion of “right” responses for the central line tilt judgment at each of the nine different line tilts in each of the two left and right square tilt conditions for each participant. We then fit each participant’s average data in each condition to two separate logistic functions with lower bounds of 0 and upper bounds of 1 using maximum likelihood estimation. To evaluate the goodness of each logistic fit, we used deviance scores of the log-likelihood ratio between a fully saturated, zero-residual model and the data model, such that deviances above the critical Chi-square indicated a significant deviation between the fit and the data (Wichmann & Hill, 2001). All deviance scores were below the critical value (χ^2^(8,0.95) = 16.92). Next, we calculated the Point of Subjective Equality (PSE) as actual line tilt necessary for each individual to perceive the central line as upright (tilted 0° from vertical) 50% of the time (the inflection point on each corresponding logistic function). Finally, we subtracted each participant’s resultant PSE in the left square tilt condition from their PSE in the right square tilt condition to calculate each participant’s measure of field dependence (PSEDif; based on a similar analysis in Corbett, et al., 2021). For both the MR and PT experiments, we calculated each participant’s average proportion of correct responses (Acc) and reaction time (RT) over all trials (MRAcc, MRRT; PTAcc; PTRT). Finally, in the RM experiment, we calculated the magnitude of the Simon Effect (SimonRT) for each participant by subtracting the average RT for correct congruent trials where the response and number stimulus were on the same side from correct incongruent trials. Importantly, we only included trials with correct responses in this analysis, as incorrect responses reversed congruency. No participant was less than 70% correct on average over all experimental trials.

*Summary Data:* Before analyzing the relative importance of the 28 predictor variables, we first examined some pertinent descriptive statistics of the categorical and continuous variables. We summarized the number of “Never,” “Sometimes,” and “Always” y-axis inverters for several key categorical variables in Table 1 to illustrate some important points about the relative distributions of inversion preferences.^6^ First, over half of participants “Always” inverted the y-axis, contrary to the distribution of inversion preferences reported in Frischmann and colleagues’ previous work (2015), where only 12 of 144 participants “Always” inverted and 16 “Sometimes” inverted. Frischmann and colleagues (2015) asked naïve participants about their inversion preferences after they were recruited to participate in the study, whereas we recruited participants for the present study from a news article specifically about inversion preferences. Therefore, the former results are more representative of inversion preferences in the general population, whereas our results are more informative about individual differences related to inversion preferences in 3rd person console games. Also, there were very few participants who inverted the x-axis, most participants were right-handed, most participants were male, and only a subset of 143 participants responded to the additional questions corresponding to the “Flight” and “Switch” measures.

**Table 1:** Summary of the number of participants who reported “Never,” “Sometimes,” and “Always” inverting the y-axis (InvertY) for each level of each categorical predictor of interest

		InvertY			
		Never (n = 40)	Sometimes (n = 34)	Always (n = 96)	Total
InvertX	Never	39	28	76	143
	Sometimes	1	4	15	20
	Always	0	2	5	7
Handed	Left	2	4	4	10
	Right	36	27	92	155
	Both	2	3	0	5
Gender	Female	4	10	8	22
	Male	36	23	85	144
	NonBinary	0	1	3	4
InvertScroll	No	23	21	58	102
	Yes	17	13	38	68
Flight	No	8	8	20	36
	Yes	21	18	68	107
	No response	11	8	8	27
Switch	No	17	8	63	88
	Yes	12	18	25	55
	No response	11	8	8	27

There were also important summary results when comparing the three types of y-axis inverters over all measured continuous variables (Table 2). The only significant main effects on y-axis inversion preference were for “Age” (*F*(2,167) = 12.13, *MSE* = 16.26, *p* < 0.001) and “YearsPlaying” (*F*(2,167) = 5.04, *MSE* = 26.95, *p* = 0.008), with older gamers who “Always” inverted having played longer than gamers who “Never” inverted (both *t*s(167) < 0.008). We did not find any of the significant differences in participants’ overall PQ (SumPresence) or ITQ (SumImmersion) scores, or on any of the ITQ sub-scales (ITQFocus, ITQInvol, ITQ Games) previously reported by Frischmann and colleagues (2015). As mentioned, our sample of participants was biased, with 56% of participants reporting that they “Always” inverted the y-axis, whereas only 12% of the more general sample of participants in Frischmann’s study reported “Always” inverting. While our study’s more equal distribution of participants over inversion preferences allows for more equated comparisons, results generalize less to the overall population.

**Table 2:** Means and standard deviations (SD) in number of participants for each of the three types of “Never,” “Sometimes,” and “Always” y-axis inverters (InvertY). Asterisks (*) in the leftmost column signify variables with significant main effects on inversion with *p* <.008

	InvertY					
	Never (n = 40)		Sometimes (n = 34)		Always (n = 96)	
	Mean	SD	Mean	SD	Mean	SD
ITQFocus	33.48	5.30	33.29	4.23	33.53	4.71
ITQInvol	27.78	6.23	30.53	7.08	29.24	7.32
ITQ Games	10.18	2.52	10.85	1.91	10.70	2.24
SumImmersion	115.88	13.60	119.41	10.91	116.80	14.00
SumPreserce	147.28	17.46	149.29	13.54	150.51	15.19
*Age	27.80	5.46	28.12	4.78	31.01	2.90
*Years Playing	16.93	5.89	17.18	5.73	19.58	4.66
MRAcc	0.80	0.17	0.85	0.15	0.85	0.14
MRRT	13.57	5.49	16.21	7.23	14.72	6.19
PTAcc	0.85	0.12	0.89	0.14	0.84	0.13
PTRT	11.27	5.17	14.31	8.86	12.43	5.45
SimonRT	0.00	0.08	0.03	0.18	0.01	0.11
PSEDif	2.47	1.79	1.88	4.02	2.22	1.85

### Predictor ranking

**Fig. 5:**
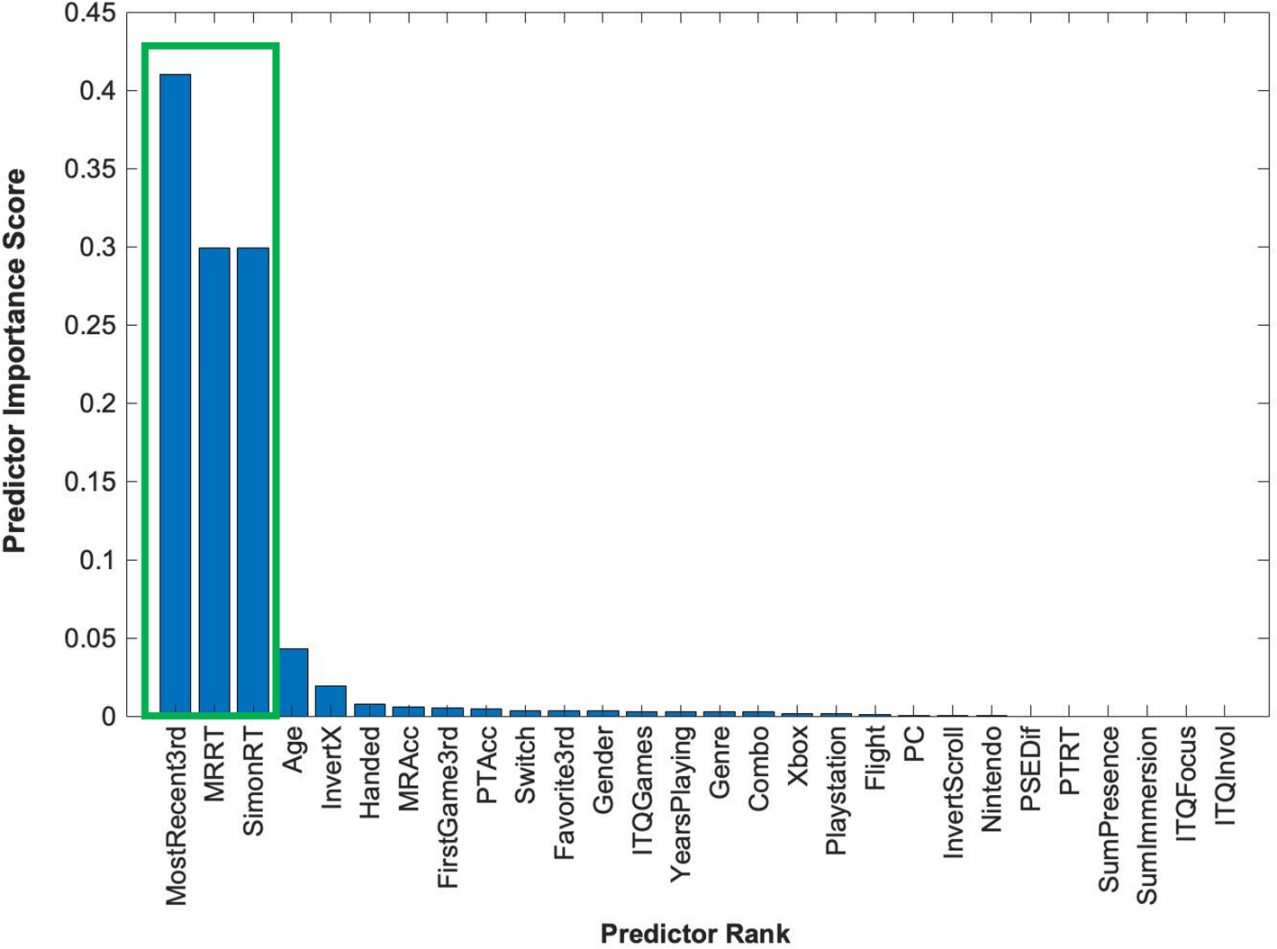
The results of the MRMR feature ranking on 28 predictors of y-axis inversion preferences. The green rectangle outlines the three top-ranked predictors

We used a Minimum Redundancy Maximum Relevance (MRMR) analysis to identify a subset of features from a larger set that maximizes the relevance to the target variable of y-axis inversion (YInvert) while minimizing redundancy among the set of selected features (Fig. 5).^7^ Instead of using variance as a criterion, MRMR measures featural relevance and redundancy based on mutual information, or the amount of information that is gained/uncertainty that is reduced about the value of one feature from knowing the value of another feature. MRMR iteratively selects features that maximize relevance and minimize redundancy. Relevance is measured as the amount of information shared between each predictor feature and the target feature. Redundancy is measured as the amount of information shared between pairs of predictor features. Because mutual information is a broader measure of the dependence between variables than covariance, it can capture both linear and nonlinear dependencies. Therefore, MRMR analysis is suitable for datasets like ours with a mix of continuous and categorical variables, and has the added benefit of discounting blank values in specific predictor estimates (e.g., missing data for participants who did not respond to the follow-up questions about flight simulator or switching inversion preferences).^8^

Instead of directly modeling the relationship between features and the target variable, MRMR ranks features by their relative informativeness (in terms of high relevance and low redundancy). Therefore, the top-ranked features should collectively cover the important aspects of the target variable without duplication. However, domain knowledge is necessary, as not all top-ranked factors may make explanatory sense. Along these lines, the first-ranked predictor, the most recent 3rd person game the participant had played (MostRecent3rd), was not informative in the context of the rankings of remaining predictors. As shown in Table 3, of the 57 total games that players listed, no single game was reported by more than nine participants and most games were reported by only a single participant.

**Table 3:** The numbers of participants who reported “Never,” “Sometimes,” and “Always” inverting the y-axis (InvertY) for each of the most recently played 3rd person games that individual participants listed (MostRecent3rd)

	**InvertY**			
	Never (n = 40)	Sometimes (n = 34)	Always (n = 96)	Total
Aer	1	0	0	**1**
APlaguesTale	0	0	1	**1**
Assassin’screed	4	2	3	**9**
Astroneer	1	0	0	**1**
Avengers	0	1	0	**1**
Batman	2	0	0	**2**
Beyond	0	0	1	**1**
Biomutant	1	0	0	**1**
Bloodborne	0	2	0	**2**
Codevein	0	1	1	**2**
Control	2	1	4	**7**
Darksouls	0	1	2	**3**
Deadbydaylight	1	0	0	**1**
DeathStranding	0	1	1	**2**
EdgeofEternity	1	0	0	**1**
Elderscrolls	1	0	0	**1**
FallGuys	2	0	1	**3**
Fallout	1	0	0	**1**
Finalfantasy	1	1	4	**6**
Fortnite	0	1	0	**1**
Gearsofwar	1	1	1	**3**
GenshinImpact	0	0	1	**1**
GhostofTsushima	2	1	2	**5**
GodofWar	1	2	2	**5**
GTA	2	2	4	**8**
Hellblade	0	1	0	**1**
Horizon	3	0	2	**5**
Immortals	0	0	1	**1**
Lifeisstrange	0	0	1	**1**
MarioOdyssey	0	0	1	**1**
Masseffect	0	1	4	**5**
Metalgear	1	0	1	**2**
Monsterhunter	1	1	1	**3**
NieR	0	1	1	**2**
NoMansSky	1	1	2	**4**
Outriders	0	0	1	**1**
Pokemon	0	0	1	**1**
Quantumbreak	1	0	0	**1**
Ratchet&clank	0	0	7	**7**
Reddeadredemption	1	1	4	**6**
Residentevil	0	0	1	**1**
Returnal	0	0	3	**3**
Rocketleague	2	0	0	**2**
Sekiro	1	0	2	**3**
Skyrim	1	0	0	**1**
Smite	1	0	0	**1**
Spiderman	0	0	2	**2**
Splatoon	0	0	1	**1**
Starwars	0	2	3	**5**
Thelastofus	0	0	6	**6**
Valheim	0	1	0	**1**
Warframe	0	0	1	**1**
Watchdogs	0	1	0	**1**
Witcher	0	0	6	**6**
XenobladeChronicles	0	1	0	**1**
Yakuza	0	0	2	**2**
Zelda	0	1	3	**4**
(blank)	3	5	11	**19**
Total	40	34	96	**170**

Furthermore, MRMR does not perform any analysis of the relationship between features and the target variable beyond assessing relevance and redundancy. Instead, it narrows down the most informative features that can be used to build and test for more effective models with fewer parameters. We used classification trees because they can model nonlinear relationships between feature and target variables for both numerical and categorical data.^9^ At each node of the classification tree, the data are partitioned based on the values of these features to best separate the classes. Comparing two classification tree models using repeated cross-validation allows for a determination of which factors significantly affect prediction accuracy. Along these lines, a classification tree for the three y-axis inversion types (Always, Sometimes, Never) trained with a model including only the second- and third-ranked predictors, mental rotation (MRRT) and response mapping (SimonRT) speeds, was significantly more accurate when compared by repeated cross-validation than a classification tree trained by a full model containing all other variables (including the most recent game played; MostRecent3rd) (*p* = 0.042). However, classification accuracy using a model trained by only MostRecent3rd was not significantly different from accuracy using a full model (*p* = 0.243), nor was classification accuracy significantly different using a model with the first three factors (MostRecent3rd, MRRT, and SimonRT) versus a full model (*p* = 0.480). Taken together, these results confirm the lack of domain-specific explanatory power for MostRecent3rd. They instead suggest gamers’ preferences may be mediated by object-based factors linked to the speed with which they could manipulate a mental image of a target, and how quickly they could correctly respond to a target as a function of the task irrelevant spatial congruency of the response.

## Discussion

Our preliminary investigation began with a literature synthesis of the factors that may predict a gamer’s preference to invert the y-axis on their gaming controller. This body of work is intended to provide an initial foundation from which to systematically pursue future empirical investigations. It also served as a starting point in our present work selecting a set of possible predictor variables we then ranked in an exploratory study. Our initial results point to the importance of object-centered cognitive factors; mental rotation and stimulus–response mapping speed. These findings are unexpected in the context of previous results that suggested y-axis inversion preferences would be related to more observer-centered processes like perspective taking^10^(e.g. Kozhevnikov, et al., 2006). It was also surprising that stimulus–response compatibility in our Simon Task (Simon & Wolf, 1963) was an important predictor, given that y-axis inversion on a gaming controller is essentially a matter of response-effect compatibility.

Our study also highlights important differences from Frischmann and colleagues’ (2015) previous work on y-axis inversion. Contrary to their findings, we did not find any effects of whether gamers Always, Sometimes, or Never inverted the y-axis as a function of presence and immersion. Importantly, our study was not specifically designed to test for differences between types of inverters on these measures, but instead to assess their predictive relationships. We also recruited participants with explicit reference to the somewhat controversial topic of y-axis inversion in gaming whereas Frischmann and colleagues (2015) recruited naïve participants. Therefore, these seemingly discrepant results are likely due to differences in the underlying designs and sampling methods.

Many participants credited previous experiences, such as playing Flight Simulator, using a specific gaming system, or their mouse/trackpad scrolling preferences as reasons for their preferences. Many also reported that their preferences had changed over time, consistent with previous reports that trackpad scrolling preferences changed with technological developments (Bury, et al., 1982; Chen & Proctor, 2013), and the switch to preferring inverted controls noted for expert versus novice laparoscopic surgeons (Bokhari, et al., 2010; Crothers, et al., 1999; Gallagher, et al., 1998; Kunde, et al., 2007). However, none of these factors had any predictive value, underscoring the need for further empirical investigations to measure behavioral effects which may not be consciously obvious to, or even reportable by users.

Taken together, findings from the reviewed literature and our exploratory results raise several important questions that merit further investigation. For starters, why is inversion more detrimental to task performance in some contexts? In contrast to many gamers’ inabilities to overcome y-axis inversion, -R-E incompatibility (fulcrum effect) is easily overcome in laparoscopic settings (Bokhari, et al., 2010; Crothers, et al., 1999; Gallagher, et al., 1998; Kunde, et al., 2007). However, in addition to inverting the camera’s y-axis as in gaming setups, the controlling instruments are also inverted about the x-axis in laparoscopic procedures. Does inverting both axes lead to different patterns of performance? There are also no studies of how gamers might perform in -S-R response mapping conditions mimicking paradoxical viewing challenges in laparoscopic surgery. Are there situations in which users can successfully adapt to new S-R mappings? Given only a handful of studies have also considered how movement goals toward or away from a target may affect performance, it is also unclear how S-E compatibility may affect different R-E and S-R scenarios.

Importantly, there are many other environmental and individual factors that differ between 3rd person console gaming and tasks like those performed in laparoscopic surgeries. As outlined, an individual’s cognitive spatial abilities may also play a role (Kozhevnikov, et al., 2006), inversion preferences can change over time (Bury, et al., 1982; Chen & Proctor, 2013), and do not necessarily transfer across domains within the same individual (Bokhari, et al., 2010; Crothers, et al., 1999; Gallagher, et al., 1998; Kunde, et al., 2007). Therefore, we cannot assume that results from one field will hold for another, necessitating investigations on a case-by-case basis. Across cases, an overarching question emerges: “How do S-R-E mappings affect performance in a given task?”.

While there is evidence of interactive relationships between S-R-E compatibilities (Kunde, et al., 2007; Müsseler & Skottke, 2011; Müsseler, et al., 2008), their specific weightings and how these may change across different situations have not yet been systematically explored. Here, we propose a modular framework for assessing performance in different S-R-E combinations. The effects of any or all of these relationships can then be assessed by developing simple tasks patterned after corresponding real-world tasks.

**Fig. 6:**
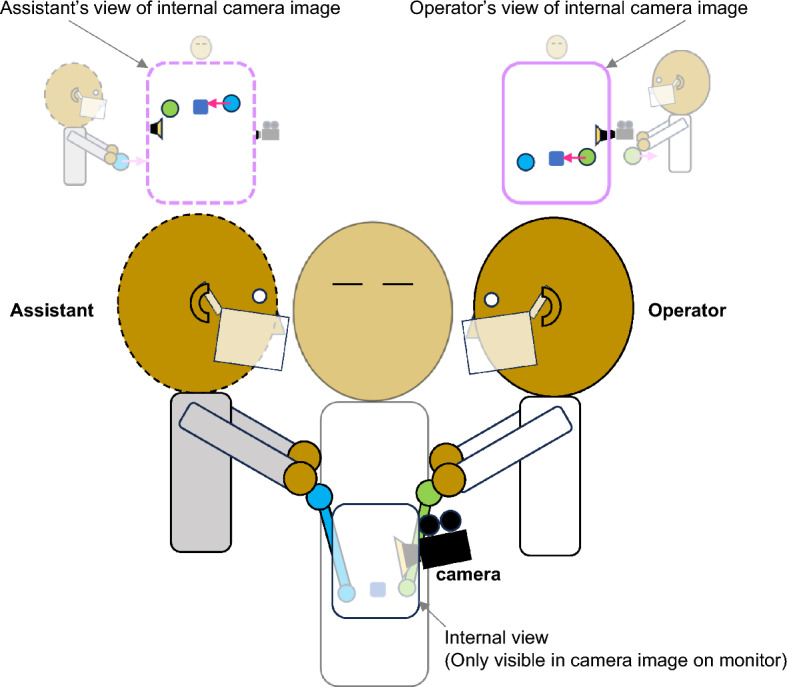
A common laparoscopic procedure with two surgeons operating on a patient from opposite sides using a camera placed internally from the operator’s perspective. For both the assistant (left) and operator (right), the controlling instruments are naturally inverted in laparoscopic procedures, such that the internal end of the instrument moves in the opposite direction as the external end of the instrument is moved in a fulcrum like manner (fulcrum effect; -R-E). The procedure is minimally invasive, but the only visual information is provided by the display from the internal camera (illustrated in each of the top panels). Without any corrections in this configuration, the operator (right) views a display that aligns with their external perspective (+ S-R), but the assistant (left) is physically positioned facing the operator (paradoxical view;—S-R)

Although some situations like gaming allow for individual customization, others are too expensive, impractical, or otherwise impossible to customize for individual users. Therefore, we chose to interpret our framework in a context abstracted from a real-world safety–critical situation where two observers must work together and may or may not be able to change display and control options. Figure 6 uses a typical set up in cholecystectomy surgery (gall bladder removal), one of the most common and challenging laparoscopic procedures. In a standard laparoscopic cholecystectomy, two surgeons (an operator and assistant) face each other on opposite sides of a patient with the operator on the patient’s left. Surgeons use separate instruments but work from a single visual display presented from the perspective of the operating surgeon. Here, we provide specific examples of how our framework can be used to make decisions in the context of a typical laparoscopic procedure. A full factorial illustration of each possible combination of S-R, R-E, and S-E in this context is publicly available as supplementary material (https://osf.io/3j6ds/).

By default, in laparoscopic surgeries, the experienced operator (Fig. 6; right) benefits from the fulcrum effect of the control scheme with the instruments moving in the opposite directions of the controls (-R-E; Bokhari, et al., 2010; Crothers, et al., 1999; Gallagher, et al., 1998; Kunde, et al., 2007) and the default camera view aligned with their own perspective (+ S-R). Some facilities are equipped for robotically-assisted procedures that could allow for the display and/or controls to be inverted from their default configurations. However, evidence is accumulating to suggest the costs outweigh the benefits (see Kalata, et al., 2023 for a recent review). Before investing in such expensive systems, our framework could be used to assess the real-world costs and benefits. For example, to assess the cost of paradoxical viewing (-S-R) for the assistant, performance in the assistant’s standard configuration (-S-R and -R-E; illustrated on the right side of Fig. 6) can be directly compared to performance in the operator’s default configuration (+ S-R and -R-E; left side of Fig. 6). Performance in these configurations can then be compared to performance when the assistant has an invertible display (+ S-R). However, if a given facility does not have the capability to present the operator and assistant with independent displays, any benefits of inverting the display for the assistant would then come at a cost to the operator (-S-R).

Furthermore, although existing results suggest that surgeons should be able to successfully adapt to a change in R-E compatibility (Hwang, et al., 2010; Johnston, et al., 2003; c.f., Müsseler, et al., 2019), any cost can be directly assessed by comparing performance in configurations when the instruments move in the same direction as the controls (+ R-E) versus when the end of the instrument moves in the opposite direction as the control (-R-E). There may also be some procedures where the costs of making errors when the goal is to avoid a given target (e.g., needing to avoid touching a delicate area) are greater than the costs of making errors by missing the target. Therefore, performance can be compared in different configurations for the operator and/or assistant when the goal is to move toward (+ S-E) versus away from a target (-S-E). A better understanding of how surgeons typically perform under fulcrum effect and paradoxical viewing conditions is also necessary for training autonomous surgical technology, such as camera guidance in collaborative surgical robotic platforms (e.g., Rivas-Blanco, et al., 2019), as well as for learning and predicting human performance to optimize interactions between AI and humans (e.g., Rivas-Blanco, et al., 2019).

Beyond these specific examples, there are a range of individual differences in how people handle discordant visual information when interacting with operating systems and how different users interact cooperatively in such situations. For example, future investigations using our framework may inform about ways to maximize performance for pilots using “real” flight consoles which can have different R-E configurations for altitude indicators (e.g., Janczyk, et al., 2015). In “moving horizon” displays, the horizon rotates in the opposite direction of controller movement (-R-E), whereas in “moving plane” displays the plane rotates in the same direction as the controller movement (+ R-E). From gamers to surgeons, understanding interactions among spatial compatibilities could allow for predictions about how easily individual users are able to accomplish a given task in a particular environment using a specific interface. It is becoming increasingly important for AI technologies to consider the possibility that users’ self-reported preferences may not always align with the best configurations for the desired patterns of performance. Our framework can surpass subjective self-reports by systematically assessing compatibility effects, and may provide a powerful tool for training AI to learn, anticipate, and cope with individual users’ behaviors.

## Conclusion

There may never be a definitive answer to the question of why some gamers invert the y-axes on their controllers. However, the broad goal of the present work was to gain insight into how a given individual’s behavioral and cognitive abilities might affect how they interact with real and virtual environments. Our initial question launched a multidisciplinary investigation into the relationships between users, interfaces, and visual displays. Understanding the factors that drive human performance is useful, if not critical for almost all aspects of gaming and virtual technologies. Being able to predict how a person will interact within a given environment can improve user experience and increase safety and efficacy. Understanding how an individual’s performance differs as a function of visual input (what is on the screen) and motor behavior (how the controller is used) may help game designers find bespoke solutions for each gamer (beyond simply inverting the y-axis) to maximize individual gamers’ experiences.

There is a gaping hole in our knowledge of how users interface with 3D environments. There are currently no academic- or industry-wide standards for assessing how the spatial relationships between the user, the controlling hardware, and the visible environment affect performance. Understanding the factors that drive human performance is useful, if not critical for almost all aspects of gaming and virtual technologies. Building on findings from previous human factors and visual perception studies, our proposed framework can be adapted and validated to assess performance costs and benefits across different combinations of user/interface/environment spatial alignments. In addition to understanding how an individual might best set up their gaming controller, this tool has great potential to save valuable time and money. A relatively quick and standardized assessment of domain-specific user performance can allow for immediate evaluation of existing configurations and optimization of developing predictive technologies.

## Open practices statement

All raw and formatted data are available in the Open Science Framework repository: https://osf.io/3j6ds/

## Supplementary Information

Additional file 1

Additional file 2

Additional file 3

## Footnotes

1 We use the term “joystick” in the context of the existing published scientific literature as synonymous to the terms “analog stick,” sometimes used in industry, and “thumb stick,” often used by gamers.

2 Note that this is not the case for VR systems. In console games, depth is created using monocular pictorial cues, and the user manipulates the view using a handheld controller. In contrast, head-mounted VR systems allow the user to move through a virtual 3D environment in real-time with additional binocular, stereoscopic depth dues, without necessitating the same options to “invert” controlling interfaces.

3 Paradoxical view is also referred to as “reverse-alignment” in some endoscopic publications.

4 Note, in the endoscopic literature, y-axis inversion actually refers to inverting the image around the left–right x-axis.

5 Note that stimuli were adjusted for screens with different aspect ratios such that stimulus dimensions remained comparable (i.e., square stimuli remained square, independent of aspect ratio and screen size).

6 Note that we did not summarize the four binary variables that coded for the individual type(s) of gaming system participants used, the aggregate “Combo” variable, or variables where participants listed specific games or selected genres because there were too many stratifications to allow for meaningful comparisons.

7 We used the fscmrmr function in MATLAB’s Statistics and Machine Learning Toolbox.

8 MRMR feature selection provides a relative ranking of predictor importance rather than an absolute scale with specific physical units. The units are more abstract and represent information content or the degree of dependence between variables rather than a concrete physical quantity.

9 We used the testckfold function in MATLAB’s Statistics and Machine Learning Toolbox.

10 Note the mental rotation task we used (Ekstrom, et al., 1976) included a visual search component. Given our results suggest mental rotation speed may play a predictive role in gamers’ inversion preferences, future studies should consider using more process-pure and fine-grained measures of mental rotation speed and accuracy as a function of angular rotation (Shepard & Metzler, 1971), and perhaps separate, contrastive measures of visual search performance.
