# Supplementary material for: Why axis inversion? Optimizing interactions between users, interfaces, and visual displays in 3D environments
Source: Cogn Res Princ Implic. 2025 Jun 23;10:33. doi: 10.1186/s41235-025-00626-5 (PMC12185817; doi:10.1186/s41235-025-00626-5)
Supplement: Supplementary file 1 — Additional file 1 [file 41235_2025_626_MOESM1_ESM.pdf]

Why-Axis Inversion: Part 1: Basic info

* Indicates required question

1. Email *

2. Participant number *

3. Your experimenter's name *

4. Today's date *

Example: January 7, 2019

5. Do you invert the y-axis? (Invert = In a 3rd person video-game, when you push the stick that controls the camera up/forward, the 'ground/floor' shows - NOT the 'sky/ceiling') *

Mark only one oval.

- Always
- Sometimes
- Never

6. Do you invert the x-axis? (Invert = In a 3rd person video-game, when you push the stick that controls the camera left, you see what is to the right of your character) *

Mark only one oval.

- Always
- Sometimes
- Never

7. How long have you been playing 3rd person console games (please input only the number of years and no other text; e.g., 12)? *

____

8. Your age *

____

9. Handedness?

Mark only one oval.

- Left
- Right
- Both equally

10. Gender (what do you identify as)? *

Mark only one oval.

- Non-binary
- Female
- Male

11. Languages spoken (please list all in which you are fluent reading, speaking, and writing)? *

Check all that apply.

- [ ] English
- [ ] Spanish
- [ ] French
- [ ] Dutch
- [ ] Portugese
- [ ] Russian
- [ ] Hindi
- [ ] Mandarin
- [ ] Arabic
- [ ] German
- [ ] Italian
- [ ] Polish
- [ ] Japanese
- [ ] Turkish
- [ ] Bengali
- [ ] Indonesian
- [ ] Other: ____________________

12. Your current console gaming system? *

____

13. The first 3rd person console game you ever played? *

____

14. Your favourite all-time 3rd person console game? *

____

15. Most recent/current 3rd person console game played? *

____

16. The last time you played this game, did you invert the y-axis? *

Mark only one oval.

- [ ] Yes
- [ ] No

17. The last time you played this game, did you invert the x-axis? *

Mark only one oval.

- [ ] Yes
- [ ] No

18. What is your scrolling preference on a laptop trackpad? *

Mark only one oval.

- [ ] When I scroll up, the page goes up
- [ ] When I scroll up, the page goes down

19. What is your favourite gaming genre (Pick one)? *

Mark only one oval.

- Action
- Adventure
- Fighting
- Platform
- Puzzle
- Racing
- Role-playing
- Shooter
- Simulation
- Sports
- Strategy
- Other: ______________

20. Why do you think people invert the y-axis? *

Part 2: Presence Questionnaire - Please answer the following questions with reference to the last time you played the current/most recent console game you listed in Part 1

21. How much were you able to control events? *

Mark only one oval.

	1	2	3	4	5	6	7	
Not								Extremely

22. How responsive was the environment to actions that you initiated (or performed)? *

Mark only one oval.

	1	2	3	4	5	6	7	
Not	◯	◯	◯	◯	◯	◯	◯	Extremely

23. How natural did your interactions with the environment seem? *

Mark only one oval.

	1	2	3	4	5	6	7	
Not	◯	◯	◯	◯	◯	◯	◯	Extremely

24. How completely were all of your senses engaged? *

Mark only one oval.

	1	2	3	4	5	6	7	
Not	◯	◯	◯	◯	◯	◯	◯	Extremely

25. How much did the visual aspects of the environment involve you? *

Mark only one oval.

	1	2	3	4	5	6	7	
Not	◯	◯	◯	◯	◯	◯	◯	Extremely

26. How much did the auditory aspects of the environment involve you? *

Mark only one oval.

	1	2	3	4	5	6	7	
Not	◯	◯	◯	◯	◯	◯	◯	Extremely

27. How natural was the mechanism which controlled movement through the environment? *

Mark only one oval.

	1	2	3	4	5	6	7	
Not	◯	◯	◯	◯	◯	◯	◯	Extremely

28. How aware were you of events occurring in the real world around you? *

Mark only one oval.

	1	2	3	4	5	6	7	
Not	◯	◯	◯	◯	◯	◯	◯	Extremely

29. How aware were you of your display and control devices? *

Mark only one oval.

	1	2	3	4	5	6	7	
Not	◯	◯	◯	◯	◯	◯	◯	Extremely

30. How compelling was your sense of objects moving through space? *

Mark only one oval.

	1	2	3	4	5	6	7	
Not	◯	◯	◯	◯	◯	◯	◯	Extremely

31. How inconsistent or disconnected was the information coming from your various senses? *

Mark only one oval.

	1	2	3	4	5	6	7	
Not	◯	◯	◯	◯	◯	◯	◯	Extremely

32. How much did your experiences in the virtual environment seem consistent with your real-world experiences? *

Mark only one oval.

	1	2	3	4	5	6	7	
Not	◯	◯	◯	◯	◯	◯	◯	Extremely

33. Were you able to anticipate what would happen next in response to the actions that you performed? *

Mark only one oval.

	1	2	3	4	5	6	7	
Not	◯	◯	◯	◯	◯	◯	◯	Extremely

34. How completely were you able to actively survey or search the environment using vision? *

Mark only one oval.

	1	2	3	4	5	6	7	
Not	◯	◯	◯	◯	◯	◯	◯	Extremely

35. How well could you identify sounds? *

Mark only one oval.

	1	2	3	4	5	6	7	
Not	◯	◯	◯	◯	◯	◯	◯	Extremely

36. How well could you localize sounds? *

Mark only one oval.

	1	2	3	4	5	6	7	
Not	◯	◯	◯	◯	◯	◯	◯	Extremely

37. How well could you actively survey or search the virtual environment using touch? *

Mark only one oval.

	1	2	3	4	5	6	7	
Not	◯	◯	◯	◯	◯	◯	◯	Extremely

38. How compelling was your sense of moving around inside the virtual environment? *

Mark only one oval.

	1	2	3	4	5	6	7	
Not	◯	◯	◯	◯	◯	◯	◯	Extremely

39. How closely were you able to examine objects? *

Mark only one oval.

	1	2	3	4	5	6	7	
Not	◯	◯	◯	◯	◯	◯	◯	Extremely

40. How well could you examine objects from multiple viewpoints? *

Mark only one oval.

	1	2	3	4	5	6	7	
Not	◯	◯	◯	◯	◯	◯	◯	Extremely

41. How well could you move or manipulate objects in the virtual environment? *

Mark only one oval.

	1	2	3	4	5	6	7	
Not	◯	◯	◯	◯	◯	◯	◯	Extremely

42. How involved were you in the virtual environment experience? *

Mark only one oval.

	1	2	3	4	5	6	7	
Not	◯	◯	◯	◯	◯	◯	◯	Extremely

43. How distracting was the control mechanism? *

Mark only one oval.

	1	2	3	4	5	6	7	
Not	◯	◯	◯	◯	◯	◯	◯	Extremely

44. How much delay did you experience between your actions and expected outcomes? *

Mark only one oval.

	1	2	3	4	5	6	7	
Not	◯	◯	◯	◯	◯	◯	◯	Extremely

45. How quickly did you adjust to the virtual environment experience? *

Mark only one oval.

	1	2	3	4	5	6	7	
Not	◯	◯	◯	◯	◯	◯	◯	Extremely

46. How proficient in moving and interacting with the virtual environment did you feel at the end of the experience? *

Mark only one oval.

	1	2	3	4	5	6	7	
Not	◯	◯	◯	◯	◯	◯	◯	Extremely

47. How much did the visual display quality interfere or distract you from performing assigned tasks or required activities? *

Mark only one oval.

	1	2	3	4	5	6	7	
Not	◯	◯	◯	◯	◯	◯	◯	Extremely

48. How much did the control devices interfere with the performance of assigned tasks or with other activities? *

Mark only one oval.

	1	2	3	4	5	6	7	
Not	◯	◯	◯	◯	◯	◯	◯	Extremely

49. How well could you concentrate on the assigned tasks or required activities rather than on the mechanisms used to perform those tasks or activities? *

Mark only one oval.

	1	2	3	4	5	6	7	
Not	◯	◯	◯	◯	◯	◯	◯	Extremely

50. Did you learn new techniques that enabled you to improve your performance? *

Mark only one oval.

	1	2	3	4	5	6	7	
Not	◯	◯	◯	◯	◯	◯	◯	Extremely

51. Were you involved in the game to the extent that you lost track of time? *

Mark only one oval.

	1	2	3	4	5	6	7	
Not	◯	◯	◯	◯	◯	◯	◯	Extremely

Part 3: Immersive Tendency Questionnaire

52. Do you ever get extremely involved in projects that are assigned to you by your boss or your instructor, to the exclusion of other tasks? *

Mark only one oval.

	1	2	3	4	5	6	7	
Nev	◯	◯	◯	◯	◯	◯	◯	Always

53. How easily can you switch your attention from the task in which you are currently involved to a new task? *

Mark only one oval.

	1	2	3	4	5	6	7	
Extr	◯	◯	◯	◯	◯	◯	◯	Extremely easy

54. How frequently do you get emotionally involved (angry, sad, or happy) in the news stories that you read or hear? *

Mark only one oval.

	1	2	3	4	5	6	7	
Nev(	◯	◯	◯	◯	◯	◯	◯	Always

55. How well do you feel today? *

Mark only one oval.

	1	2	3	4	5	6	7	
Terr	◯	◯	◯	◯	◯	◯	◯	Terrific

56. Do you easily become deeply involved in movies or TV dramas? *

Mark only one oval.

	1	2	3	4	5	6	7	
Not	◯	◯	◯	◯	◯	◯	◯	Extremely

57. Do you ever become so involved in a television program or book that people have problems getting your attention? *

Mark only one oval.

	1	2	3	4	5	6	7	
Nev(	◯	◯	◯	◯	◯	◯	◯	Always

58. How mentally alert do you feel at the present time? *

Mark only one oval.

	1	2	3	4	5	6	7	
Not	◯	◯	◯	◯	◯	◯	◯	Extremely

59. Do you ever become so involved in a movie that you are not aware of things happening around you? *

Mark only one oval.

	1	2	3	4	5	6	7	
Nev	◯	◯	◯	◯	◯	◯	◯	Always

60. How frequently do you find yourself closely identifying with the characters in a story line? *

Mark only one oval.

	1	2	3	4	5	6	7	
Nev	◯	◯	◯	◯	◯	◯	◯	Always

61. Do you ever become so involved in a video game that it is as if you are inside the game rather than moving a joystick and watching the screen? *

Mark only one oval.

	1	2	3	4	5	6	7	
Nev	◯	◯	◯	◯	◯	◯	◯	Always

62. On average, how many books do you read for enjoyment in a month (just enter a number)? *

63. What kind of books do you read most frequently? *

Mark only one oval.

- Spy novels
- Adventure
- Westerns
- Biographies/Autobiographies
- Fantasies
- Romance
- Mysteries
- Science fiction
- History
- Other fiction
- Other non-fiction

64. How physically fit do you feel today? *

Mark only one oval.

	1	2	3	4	5	6	7	
Not								Extremely

65. How good are you at blocking out external distractions when you are involved in something? *

Mark only one oval.

	1	2	3	4	5	6	7	
Terr	◯	◯	◯	◯	◯	◯	◯	Terrific

66. When watching sports, do you ever become so involved in the game that you react as if you were one of the players? *

Mark only one oval.

	1	2	3	4	5	6	7	
Nev	◯	◯	◯	◯	◯	◯	◯	Always

67. Do you ever become so involved in a daydream that you are not aware of things happening around you? *

Mark only one oval.

	1	2	3	4	5	6	7	
Nev	◯	◯	◯	◯	◯	◯	◯	Always

68. Do you ever have dreams that are so real that you feel disoriented when you awake? *

Mark only one oval.

	1	2	3	4	5	6	7	
Nev	◯	◯	◯	◯	◯	◯	◯	Always

69. When playing sports, do you become so involved in the game that you lose track of time? *

Mark only one oval.

	1	2	3	4	5	6	7	
Nev	◯	◯	◯	◯	◯	◯	◯	Always

70. Are you easily disturbed when working on a task? *

Mark only one oval.

	1	2	3	4	5	6	7	
Nev	◯	◯	◯	◯	◯	◯	◯	Always

71. How well do you concentrate on enjoyable activities? *

Mark only one oval.

	1	2	3	4	5	6	7	
Terr	◯	◯	◯	◯	◯	◯	◯	Terrific

72. How often do you play arcade or video games? (often should be taken to mean every day or every two days, on average.) *

Mark only one oval.

	1	2	3	4	5	6	7	
Nev	◯	◯	◯	◯	◯	◯	◯	Often

73. How well do you concentrate on disagreeable tasks? *

Mark only one oval.

	1	2	3	4	5	6	7	
Terr	◯	◯	◯	◯	◯	◯	◯	Terrific

74. Have you ever gotten excited during a chase or fight scene on TV or in the movies? *

Mark only one oval.

	1	2	3	4	5	6	7	
Nev	◯	◯	◯	◯	◯	◯	◯	Always

75. To what extent have you dwelled on personal problems in the last 48 hours? *

Mark only one oval.

	1	2	3	4	5	6	7	
Not	◯	◯	◯	◯	◯	◯	◯	Constantly

76. Have you ever gotten scared by something happening on a TV show or in a movie? *

Mark only one oval.

	1	2	3	4	5	6	7	
Nev	◯	◯	◯	◯	◯	◯	◯	Always

77. Have you ever remained apprehensive or fearful long after watching a scary movie? *

Mark only one oval.

	1	2	3	4	5	6	7	
Nev	◯	◯	◯	◯	◯	◯	◯	Always

78. Do you ever avoid carnival or fairground rides because they are too scary? *

Mark only one oval.

	1	2	3	4	5	6	7	
Nev	◯	◯	◯	◯	◯	◯	◯	Always

79. How frequently do you watch TV soap operas or docu-dramas? *

Mark only one oval.

	1	2	3	4	5	6	7	
Nev	◯	◯	◯	◯	◯	◯	◯	Always

80. Do you ever become so involved in doing something that you lose all track of time? *

Mark only one oval.

	1	2	3	4	5	6	7	
Nev	◯	◯	◯	◯	◯	◯	◯	Always

This content is neither created nor endorsed by Google.

Google Forms
