# Supplementary material for: Why axis inversion? Optimizing interactions between users, interfaces, and visual displays in 3D environments
Source: Cogn Res Princ Implic. 2025 Jun 23;10:33. doi: 10.1186/s41235-025-00626-5 (PMC12185817; doi:10.1186/s41235-025-00626-5)
Supplement: Supplementary file 3 — Additional file 3 [file 41235_2025_626_MOESM3_ESM.pdf]

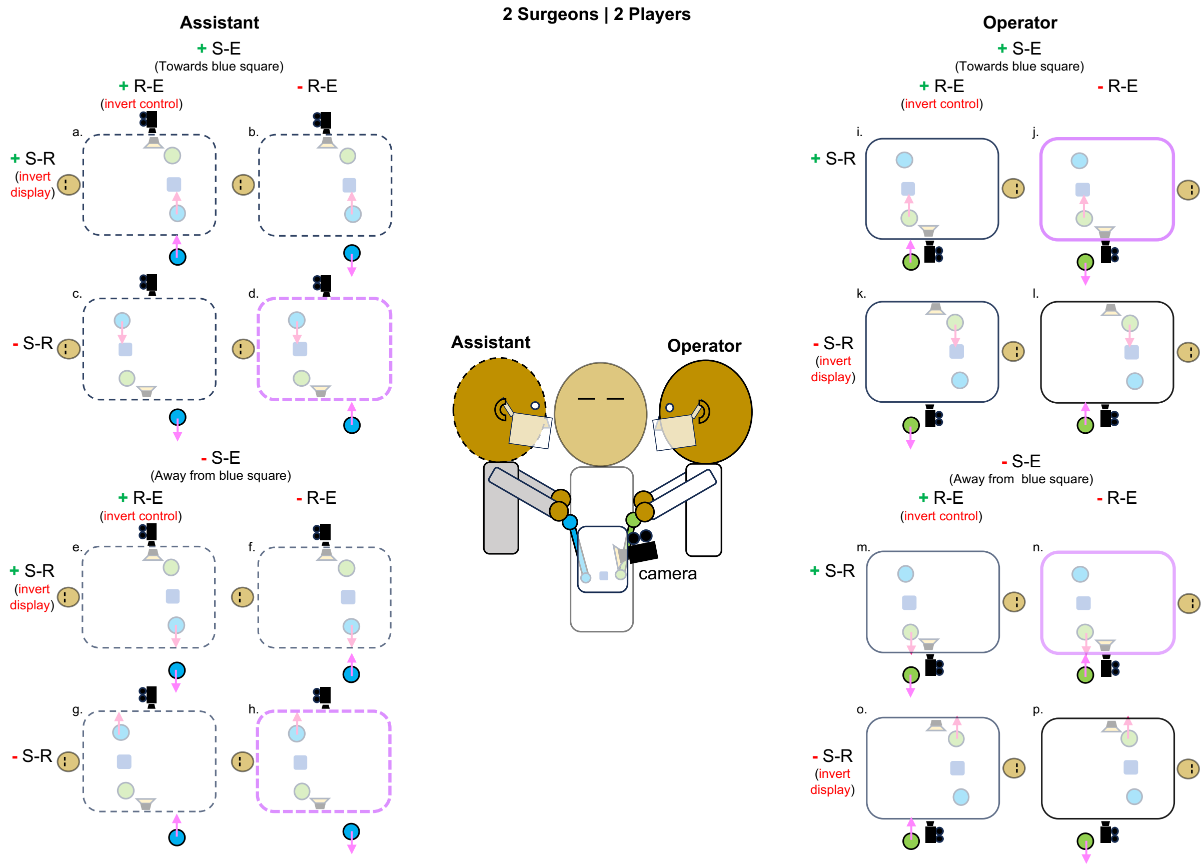

S-R-E Framework

All factorial S-R, R-E, and S-E compatibility combinations illustrated in the context of a common colorectal laparoscopic procedure with two surgeons operating on a patient from opposite sides using a camera placed internally from the operator's perspective. The procedure is minimally invasive, but the only visual information is provided by the display from the internal camera (illustrated in each of the semi-transparent panels).

The operator's possible display and control configurations (solid lines) are presented on the right half of the figure, and the assistant's configurations (dashed lines) are displayed on the left. Blue and green circles correspond to the assistant and operator's same-color controlling instruments. The default display and control configurations for both assistant (dashed) and operator (solid) are outlined in violet. In each of the 16 possible configurations, the patient's head, the external portion of the camera, and the internal camera position are provided as reference points. The direction the surgeon moves the controlling instrument outside of the patient relative to their own body is indicated by bright magenta arrows, and the direction the controlling instrument moves inside the patient's body relative to the blue square is indicated by semi-opaque pink arrows.

S-R Compatibility: Without any corrections in this configuration, the assistant's view of the display is as if looking from the operator's perspective, but the assistant is physically positioned facing the operator (paradoxical view). "Invert display" in red text refers to converting the native view provided by the camera to a horizontal/vertical reversed image on the display seen by the surgeon.

R-E Compatibility: The controlling instruments are naturally inverted in laparoscopic procedures, such that the internal end of the instrument moves in the opposite direction as the external end of the instrument is moved in a fulcrum like manner (fulcrum effect). Therefore, "Invert control" refers to changing from the native (inverted) instrument setting.

S-E Compatibility: The top four panels on each left and right side of the display illustrate cases where the user is tasked to move towards the stimulus, whereas the bottom four panels on each side illustrate situations in which the user must move away from the stimulus.

a). +S-E +S-R, +R-E for the assistant when tasked to move towards the blue square while viewing an inverted display that aligns with their external perspective and a controlling instrument that is inverted to move the same direction internally as they move it externally. b). +S-E +S-R, -R-E for the assistant when tasked to move towards the blue square while viewing an inverted display that aligns with their external perspective and a controlling instrument that moves the opposite direction internally as they move it externally. c). +S-E -S-R, +R-E for the assistant when tasked to move towards the blue square while viewing a display that is the opposite of their external perspective and a controlling instrument that is inverted to move the same direction internally as they move it externally. d). +S-E -S-R, -R-E (default configuration) for the assistant when tasked to move towards the blue square while viewing a display that is the opposite of their external perspective and a controlling instrument that moves the opposite direction internally as they move it externally.

e). -S-E +S-R, +R-E for the assistant when tasked to move away from the blue square while viewing an inverted display that aligns with their external perspective and a controlling instrument that is inverted to move the same direction internally as they move it externally. f). -S-E +S-R, -R-E for the assistant when tasked to move away from the blue square while viewing an inverted display that aligns with their external perspective and a controlling instrument that moves the opposite direction internally as they move it externally. g). -S-E -S-R, +R-E for the assistant when tasked to move away from the blue square while viewing a display that is the opposite of their external perspective and a controlling instrument that is inverted to move the same direction internally as they move it externally. h). -S-E -S-R, -R-E (default configuration) for the assistant when tasked to move away from the blue square while viewing a display that is the opposite of their external perspective and a controlling instrument that moves the opposite direction internally as they move it externally.

i). +S-E +S-R, +R-E for the operator when tasked to move towards the blue square while viewing a display that aligns with their external perspective and a controlling instrument that is inverted to move the same direction internally as they move it externally. j). +S-E +S-R, -R-E (default configuration) for the operator when tasked to move towards the blue square while viewing a display that aligns with their external perspective and a controlling instrument that moves the opposite direction internally as they move it externally. k). +S-E -S-R, +R-E for the operator when tasked to move towards the blue square while viewing an inverted display that is the opposite of their external perspective and a controlling instrument that is inverted to move the same direction internally as they move it externally. l). +S-E -S-R, -R-E for the operator when tasked to move towards the blue square while viewing an inverted display that is the opposite of their external perspective and a controlling instrument that moves the opposite direction internally as they move it externally.

m). -S-E +S-R, +R-E for the operator when tasked to move away from the blue square while viewing a display that aligns with their external perspective and a controlling instrument that is inverted to move the same direction internally as they move it externally. n). -S-E +S-R, -R-E (default configuration) for the operator when tasked to move away from the blue square while viewing a display that aligns with their external perspective and a controlling instrument that moves the opposite direction internally as they move it externally. o). -S-E -S-R, +R-E for the operator when tasked to move away from the blue square while viewing an inverted display that is the opposite of their external perspective and a controlling instrument that is inverted to move the same direction internally as they move it externally. p). -S-E -S-R, -R-E for the operator when tasked to move away from the blue square while viewing an inverted display that is the opposite of their external perspective and a controlling instrument that moves the opposite direction internally as they move it externally.

Note that the illustration is similar to a task for a laparoscopic training procedure, but there are many important additional considerations when experts perform the actual corresponding procedures. In general, we assume an unobstructed, top-down camera vantage that is far from the shadowed, occluded, and limited field of view imagery surgeons must delicately navigate.
